# Quad-port MIMO antenna with high isolation characteristics for sub 6-GHz 5G NR communication

**DOI:** 10.1038/s41598-023-46413-4

**Published:** 2023-11-04

**Authors:** Trushit Upadhyaya, Vishal Sorathiya, Samah Al-shathri, Walid El-Shafai, Upesh Patel, Killol Vishnuprasad Pandya, Ammar Armghan

**Affiliations:** 1 Electronics and Communication Department, Chandubhai S. Patel Institute of Technology, Charotar University of Science and Technology (CHARUSAT), Changa, 388421 India; 2 Faculty of Engineering and Technology, Parul Institute of Engineering and Technology, Parul University, Waghodia Road, Vadodara, 391760 India; 3 Department of Information Technology, College of Computer and Information Sciences, Princess Nourah bint Abdulrahman University, P.O. Box 84428, 11671 Riyadh, Saudi Arabia; 4 Security Engineering Lab, Computer Science Department, Prince Sultan University, 11586 Riyadh, Saudi Arabia; 5 Department of Electronics and Electrical Communications Engineering, Faculty of Electronic Engineering, Menoufia University, Menouf, 32952 Egypt; 6 Department of Electrical Engineering, College of Engineering, Jouf University, 72388 Sakaka, Saudi Arabia

**Keywords:** Electrical and electronic engineering, Techniques and instrumentation

## Abstract

A four-port MIMO antenna with high isolation is presented. The antenna is primarily envisioned to cover the n48 band of Frequency Range-1 (FR-1) with TDD duplex mode. The engineered antenna has electrical dimensions of 90 × 90 × 1.57 mm^3^. The size miniaturization of a single antenna unit is achieved through an optimized placement of slots and extended arms. The quad-antennas are then placed orthogonally to achieve antenna diversity. The antenna resonates at 3.56 GHz and 5.28 GHz having 2:1 VSWR fractional bandwidth of 1.82% and 2.12%. The proposed resonator provides 88.34% and 79.28% efficiency at lower and upper bands, respectively. The antenna is an exceptional radiator regarding MIMO diversity performance owing to high inter-element isolation. The values of envelope correlation coefficient < 0.05, channel capacity loss is nearly 0.1 bits/sec/Hz, and total active reflection coefficient is − 24.26. The full ground plane profile aids in high directivity and cross-pol isolation. The antenna exhibits a gain of 4.2 dBi and 2.8 dBi, respectively, justifying intended application requirements. There is a good coherence between simulation and experimental results. The self-decoupled antenna poses its application in 5G and WLAN Communication Applications.

## Introduction

The antenna is a vital component of the communication system. The data communication requirement continuously evolves about data transmission increase^1^. Owing to such demand, antenna performance enhancement seeks continuous research and development^2^. High-speed data rate is a key demand for emerging technologies such as virtual reality, online gaming, and smart city^3^. The multiple antenna structural designs allow high data processing and throughputs to be available using advanced radio propagation schemes. The 5G new radio (nr) technology, a unified and capable wireless air interface, envisions improved reliability and extremely high data rate^4^. 5G NR supports adaptive bandwidth support, less time lag and minimal network losses. The carriers can be simultaneously aggregated over the spectrum. With 5G momentum, the communication networks shall provide end-to-end connectivity to user equipment directly connected to the 5G core communication network. Multi-network connectivity shall enable users to achieve the Gigabit per second data rate^5^. The 4G services do not have the potential to cater for the requirement of data traffic created through enormously demanding applications such as live video streaming, cloud services, and online gaming, to name a few^6^. The 5G technology is now being implemented by the majority of nations across the globe to accomplish the need to handle large data^7^. The centimetre and millimetre wave spectrum of 3–300 GHz has been quite effectively utilized for 5G communication technology for achieving extremely high throughput in the order of some Gigabit per second. The additional benefit of targeting this frequency is that a lower spectrum is utilized in parallel for other communication applications such as ISM, Wi-Fi and WiMAX. In contrast, a higher spectrum can be available for 5G wireless communication applications.

The multiple-input multiple-output (MIMO) technology enables enhanced channel capacity and moderates multipath fading^8^. The optimal spectrum efficiency and resource sharing in channel allocation enable more end-user devices^9^. The radio propagation issue of fading is addressed as each antenna faces inconsistent multipath fading and gets nullified. A wide range of multiplexing techniques exist to provide a large degree of freedom in 5G technology^10^. The MIMO technology significantly reduces radio propagation issues through multiple transmit and receive antennas, multiplexing techniques, and high spectrum efficiency. The MIMO antennas should be able to provide high inter-element isolation between antenna elements and gain^11–13^. The high inter-element isolation aids in achieving higher throughput from the MIMO antenna system causing better communication efficiency^14–16^. The MIMO antennas can exhibit increased channel capacity and enhanced link reliability through multipath propagation techniques. The MIMO antennas having high inter-element isolation is a key design challenge for antenna designers, especially with a closely packed device environment^14,17^. The mutual coupling between the MIMO antenna elements primarily causes E-plane radiation to deteriorate due to surface current flows in the neighbor element. However, H-plane would not get significantly affected due to magnetic coupling through the air^18^. In addition, the wideband antenna shall suffer higher if the mutual coupling between elements is high. It is typically suggested that the inter-element distance should be at least half-wavelength to counter issues that emerge through mutual coupling. The arrangement of antennas for MIMO shall have to deal with the tradeoff between mutual coupling and antenna radiation characteristics. A good MIMO characteristic shall need at least mutual coupling isolation of 20 dB. There are several decoupling mechanisms. A significant quantum of research is present in the literature for improving the antenna isolation characteristics^12,19–22^. The presented antenna does not employ any additional decoupling mechanism for improving the isolation characteristics. State-of-the-art research in MIMO antenna design has been recently reported^23,24^. The literature highlighting bandwidth and gain enhancement techniques for MIMO antenna can be found in^25–30^.

The patch antenna provides ease in integration and manufacturing. Mobile communication antennas significantly suffer from space-constrained environments. The surface mountable antennas provide viability of the placement in such conditions. The patch antennas usually suffer from less bandwidth and moderate gain issues because of the dielectric material loss at high frequencies above 1 GHz. The presented study focused on high inter-element isolation, good antenna gain and improved MIMO characteristics. A quad-element MIMO antenna is aligned orthogonally to contribute to targeted applications to achieve the desired antenna parameters. The radiating elements of planar resonators are kept in anti-parallel mode to improve diversity parameters significantly. The antenna presents its usage in NR FR-1 5G communication and WLAN applications. The novelties of the structure achieved in this manuscript are as follows:

- A novel antenna design structure which was designed with the different iteration of the shape to achieve the desired frequency band of operation.
- Proposed MIMO antenna designed by engraving the single side of the PCB where the ground plane does not require the engraving process. This will reduce the overall fabrication cycle for the antenna.
- The values of the cross-port isolation are better than 15 dB which provides good isolation between antenna elements. Hence, due to the excellent inter-element isolation, the antenna performs very well as a radiator regarding MIMO diversity performance.
- This structure offers the dual band of the operation with maximum of 4.2 dBi of the gain 2.8 GHz of the bandwidth.
- The antenna structure and the different approach can help to design the frequency selective dual band antenna. Our antenna design approach helps to identify the resonating frequency bands between 5.2 to 5.6 GHz where the first frequency band are almost same in all the iteration.

## Design and geometric configuration

**Figure 1:**
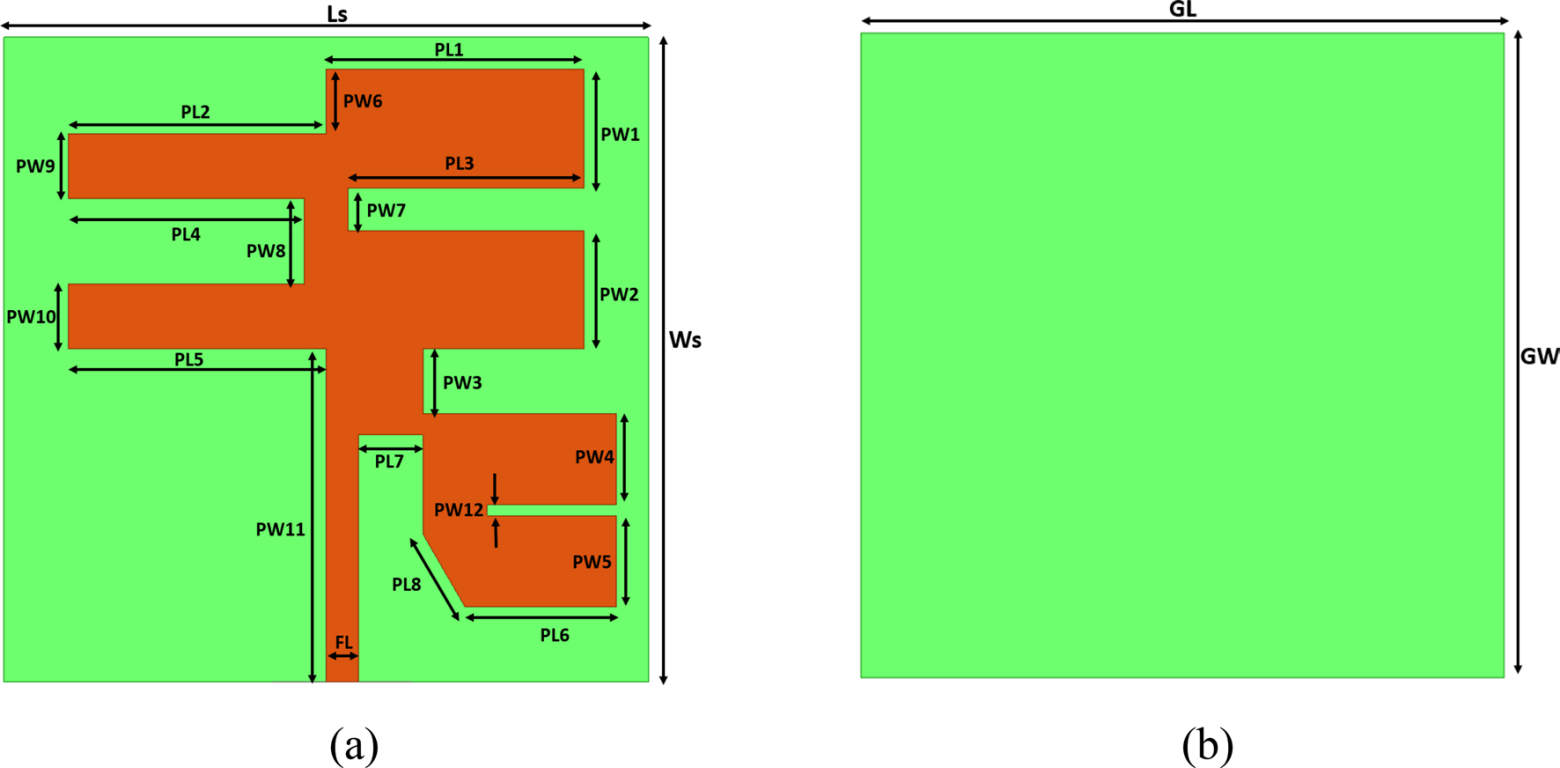
Schematic of the antenna (**a**) Top view with a conductive patch of the single-element antenna. (**b**) Fully ground plane with a conductive plane.

The proposed quad-element MIMO antenna is designed on AD255C Rogers laminate. The AD255 has low-lossy characteristics with a loss tangent of 0.0014 and a dielectric constant 2.55. It has a very high copper peel strength of more than 10  pli, a good coefficient of thermal expansion in order 34 ppm/°C, a strong tensile strength of 55.8 MPa and radiating element copper profile of 35 oz. The geometric design of the proposed single-unit antenna is exhibited in Fig. 1, with its physical parameters shown in Table 1. The antenna was simulated in a finite element solver high-frequency structure simulator. The antenna has the mechanical dimensions of 90 × 90 × 1.57 mm^3^ or the electrical dimensions of 1.06 λ × 1.06 λ × 0018 λ at a lower frequency. The quality of a patch antenna can be increased in several different ways: by using a thick substrate, cutting a resonant slot inside the patch, using a low dielectric substrate, configuring multiple resonators in a stack, using different impedance matching and feeding methods, and by employing slot antenna geometry. Improving one attribute often worsens the other; this is especially true when it comes to antenna bandwidth and size, and therefore the careful approach to design evolution with necessary computational checks at every step is required in antenna design.

**Table 1:** Antenna physical dimensions.

Parameter	Size (in mm)	Parameter	Size (in mm)
Ls	30	PW1	5.5
Ws	30	PW2	5.5
PL1	12	PW3	3
PL2	12	PW4	4.25
PL3	11	PW5	4.25
PL4	11	PW6	3
PL5	12	PW7	2
PL6	7.03	PW8	4
PL7	3	PW9	3
PL8	3.92	PW10	3
FL	1.5	PW11	15.5

**Figure 2:**
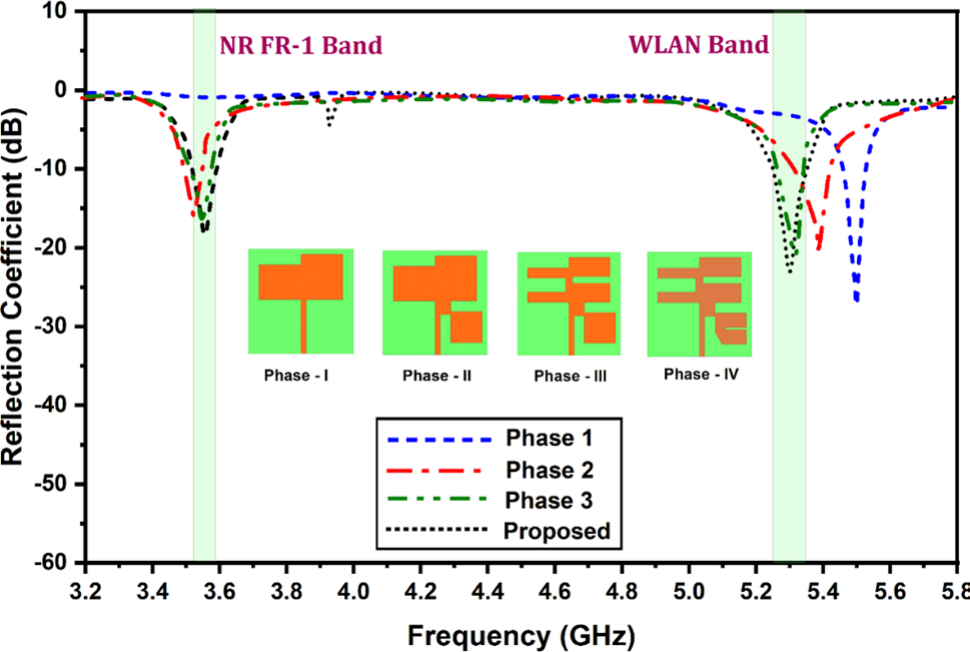
Reflection coefficient for the different phases of the unit cell antenna structure.

The proposed antennas have undergone four phases of development, and the fourth and final phase has been demonstrated to produce the desired results. Phase 1 consists solely of the carved-out rectangular portion for improved bandwidth and s-parameters. In the second phase, square sections were added to the bottom right corner of the carved-out rectangular patch, as optimal reflection results were not quite attained. The phase 2 S_11_ results improved, but the resonance peak did not occur in the desired wireless frequency. After considering and reviewing a mountain of literature on sliding the resonant peak by carving out the patches in microstrip patch antenna, and thus more carving out occurring like a driven element in the yagi-uda antenna, the width of the deformed rectangular patch was kept wider on the right and narrower on the left. This concept successfully produced the desired s parameters, and its resonating point was in the NR FR1 and WLAN bands. To fine-tune and optimize the design, dividing the square area into wider elements was determined to achieve optimized results in the preferable frequency band. For all four phases of the design, the bottom view of the antenna states that the full ground plane of the metal has been placed with no further modifications. Figure 2 describes the graphical representation of the evolution of the proposed MIMO antenna structure with the detailed S_11_ parameters.

In antennas, the significance of the S_11_ parameter rests in its capacity to measure the quantity of power reflected by the transmitter from the antenna. This parameter denotes the degree to which the antenna and transmission line or source impedance are matched. A low S_11_ value indicates a good impedance match, which means that most of the power is transmitted from the transmitter to the antenna, and very little is reflected. A high S_11_ value, on the other hand, signifies an impedance mismatch that results in a significant quantity of power being reflected in the transmitter. MIMO systems require a strong impedance match for efficient power transfer and signal integrity. When the S_11_ parameter is small, it ensures that the majority of the transmitted power is effectively radiated by the antenna instead of being squandered as reflected power. This results in increased signal strength, decreased interference, and enhanced overall system performance.

In addition, the S_11_ parameter aids in determining the antenna's operating bandwidth. Analyzing the S_11_ characteristics across a range of frequencies makes it feasible to identify the frequency bands in which the return loss is acceptable. This information facilitates the selection of appropriate frequency bands for MIMO operation and the design of filters or other components to enhance antenna performance within those frequency bands. The S_11_ parameter contributes to the fine-tuning of patch antenna design. Adjustments to the antenna size, substrate type, feeding structure, and other factors may be made using the S_11_ values to optimize performance and obtain desired features such as resonance frequency, radiation pattern, and gain, and Fig. 2 is the perfect way to describe how the reflection coefficient vs frequency results have helped the design process to get the optimized antenna design.

The relationship between S_11_ and frequency for all four phases is illustrated in Fig. 2. In phase 1, the S_11_ is nearly 0 dB across the entire band, with one good resonance of approximately − 30 dB outside any of the wireless frequencies we are concentrating on. Phase 2 yields improved reflection coefficient results, but not in the desired wireless frequencies. Figure 2 demonstrates that, as of phase 3, acceptable levels of S_11_ resonance are occurring in the desired wireless frequencies. Though a − 20 dB resonance is less desirable than a − 30 dB resonance, some of the applications in these wireless bands where moderate levels of reflections are tolerable for IoT devices, non-critical wireless communication systems, home Wi-Fi networks, low-power wireless sensor networks, and indoor communication networks so long as the system provides adequate communication quality.

**Figure 3:**
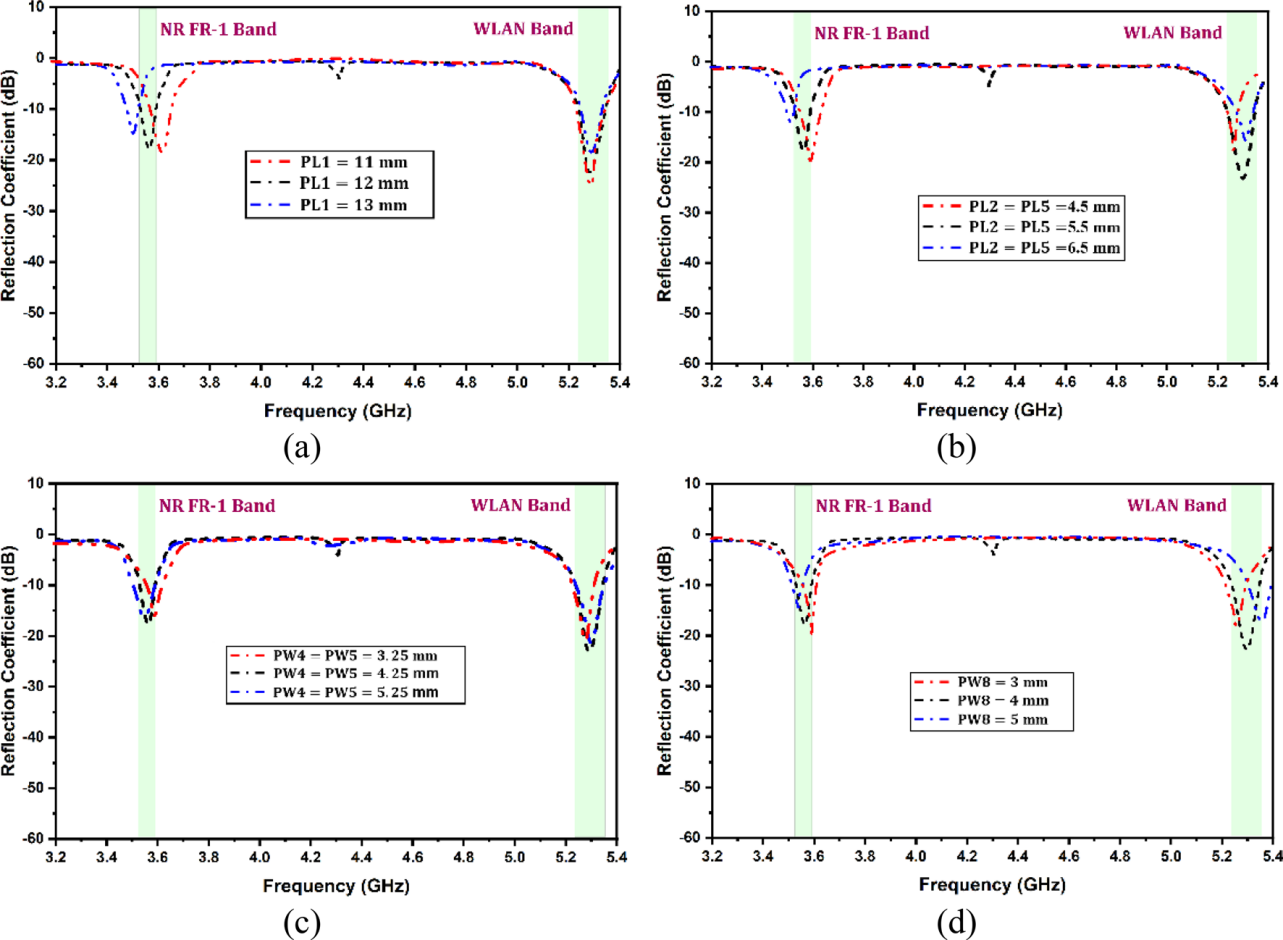
Variation in the reflectance coefficient for the different antenna design parameters (**a**) PL1, (**b**) PL2, (**c**) PW4, and (**d**) PW8.

A parametric study was conducted to enhance an antenna's design and optimization processes while addressing the issue of resonant frequency and improving its performance in terms of return loss. The study examined various parameters, including slot lengths and widths, to determine their impact on the antenna's return loss and impedance bandwidth. To better understand the effects of changing parameters on the antenna’s performance, only one parameter was altered at a time while keeping the others constant. Several parameters influence the resonant frequencies and matching of the antenna and Fig. 3 demonstrates the effectiveness of these parameters on resonant frequency and return loss. Figure 3 is the return loss of the phase 4 antenna as a function of frequency for different values of design parameters, such as slot lengths and widths of different slots.

All the parameters in a patch antenna control the properties of the antenna, such as length, width, height, and permittivity. Here, a parametric study of the length and width of different slots is given as properties such as resonant frequency, input impedance, return loss, and radiation patterns were the focus of this MIMO antenna structure. The slots length and not the width heavily influence the resonant frequency, while the relationship between the length of the slot and resonant frequency can be driven as per Eq. (1):

1 $$ {\text{f}}_{{\text{c}}} \approx \frac{{\text{c}}}{{2{\text{L}}\sqrt {{\upvarepsilon }_{{\text{r}}} } }} = \frac{1}{{2{\text{L}}\sqrt {{\upvarepsilon }_{0} {\upvarepsilon }_{{\text{r}}} {\upmu }_{0} } }} $$

The width of the slots affects the input impedance and radiation patterns of the antenna. It has been theorized that the wider the patch, the lower the input impedance. The slot's width predominantly determines the coupling between the slot antenna and electromagnetic radiation. A narrower slot width enhances the intensity of the electric field within the slot, resulting in a greater input impedance. It is because a narrower slot restricts the passage of currents along the slot margins, resulting in a greater concentration of electric field and a greater impedance.

On the other hand, the slot length influences the antenna's resonant frequency. The length of the slot, which functions as a resonator, determines the wavelength at which the antenna emits or receives electromagnetic radiation efficiently. The antenna can be tuned to resonate at various frequencies by altering the slot length. In contrast to the slot width, the effect of slot length on input impedance is relatively minor. The relation between width and impedance for the rectangular-like patch can be approximated by given Eq. (2).

2 $$ {\text{Z}}_{{\text{in }}} = 90\left( {\frac{{{\upvarepsilon }_{{\text{r}}}^{2} }}{{{\upvarepsilon }_{{\text{r}}} - 1}}} \right)\left( {\frac{{\text{L}}}{{\text{W}}}} \right)^{2} $$

Input impedance is directly associated with the return loss. The width parameter is heavily effective in improving input impedance and, thereby, return loss because when the slot width of an antenna is increased, the effective aperture of the antenna also increases. This results in the antenna capturing more electromagnetic energy, which leads to higher power reception. Consequently, the reflected power decreases, and the return loss improves. It means that increasing the slot width usually leads to a reduction in the magnitude of the reflected power and an improvement in the return loss.

On the other hand, decreasing the slot width of an antenna results in a decrease in the effective aperture of the antenna, leading to lower power reception. It can cause more energy to be reflected, increasing the magnitude of the reflected power and worsening the return loss. Figure 3a and b show that the change in slots length is directly associated with a resonant frequency, and by increasing the length, the frequency decreases, but we have to decrease the length in such a way that both the bands the resonant frequency lies within the limit. Figure 3c and d demonstrate that the return loss is directly related to the width of the antenna slot. However, it is important to note that other factors, such as the feed position and substrate, can also significantly impact the return loss. In a MIMO design, it is crucial to thoroughly analyse the feeding position of all four components before proceeding with the fabrication step. It will ensure the design is optimized for maximum performance and minimal return loss. This study concludes that it is imperative to recognize that its dimensions do not exclusively govern the performance of a slot antenna. Other factors, such as substrate properties and dielectric constants, can also influence its performance.

**Figure 4:**
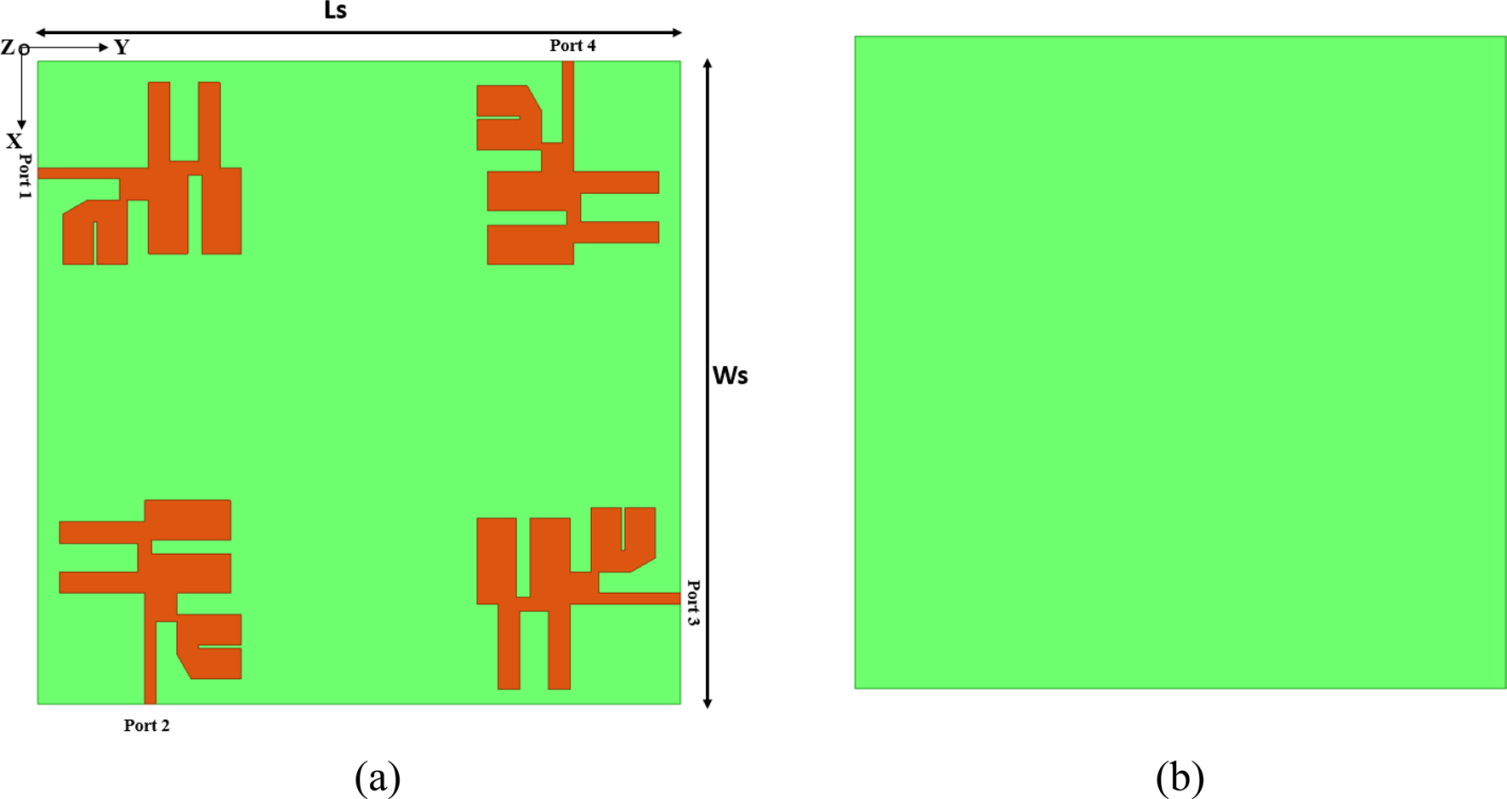
Schematic of the four-element MIMO antenna structure with (**a**) top view and (**b**) bottom view.

Furthermore, modifications made to the slot width or length can have a cascading effect on other antenna parameters, such as bandwidth, radiation pattern, and efficiency. Thus, it is essential to consider all pertinent parameters and their interrelationships when designing a slot antenna to ensure optimal performance. The proposed resonator design employs the four MIMO elements placed orthogonally at the corner of the top layer, as illustrated in Fig. 4a. In a MIMO (Multiple-Input Multiple-Output) antenna system, utilising multiple antenna elements enables spatial multiplexing, diversity, or beamforming techniques, which can significantly enhance the capacity and performance of wireless communication systems.

A common configuration in MIMO systems involves placing four antenna elements at the four corners in an orthogonal position. Placing the antenna elements at the four corners in an orthogonal position is advantageous, enabling spatial separation and maximizing the spatial diversity gain. Positioning the antennas at different corners allows the signals to undergo varying propagation paths and fading conditions, ultimately improving the overall system performance. The orthogonal placement of the antenna elements involves arranging them so that their polarization or radiation patterns are orthogonal to each other. This arrangement allows for more independent spatial channels and reduces interference between the antenna elements.

**Figure 5:**
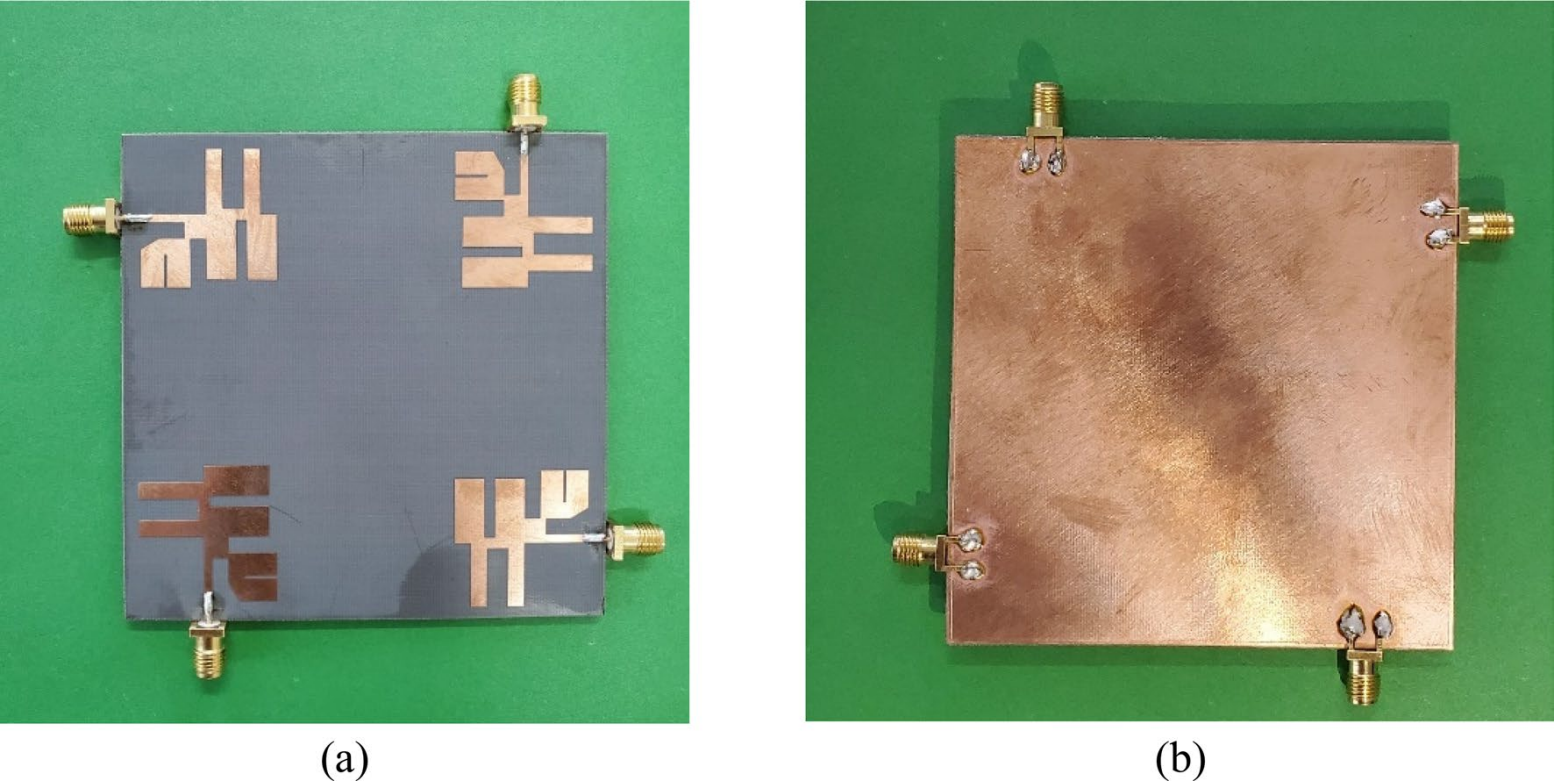
Fabricated prototype of the four-element MIMO antenna structure.

**Figure 6:**
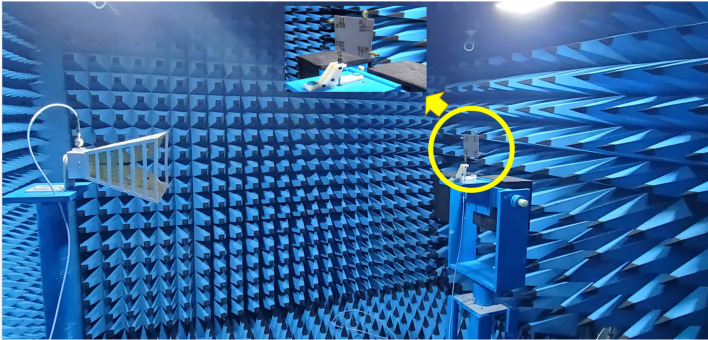
Antenna measurement setup in anechoic chamber.

Consequently, it enables higher data rates and improves the communication reliability of the system. The proposed resonator design employs the four MIMO elements placed orthogonally at the corner of the top layer, as illustrated in Fig. 4b. The fabricated prototype of the antenna is shown in Fig. 5. The structure is fabricated with four elements of the proposed MIMO antenna, and results of the return loss, directivity and other MIMO parameters are compared with simulated results. The prototype was measured in anechoic chamber environment as shown in Fig. 6. The setup incorporates load termination of 50 Ω for remaining ports.

**Figure 7:**
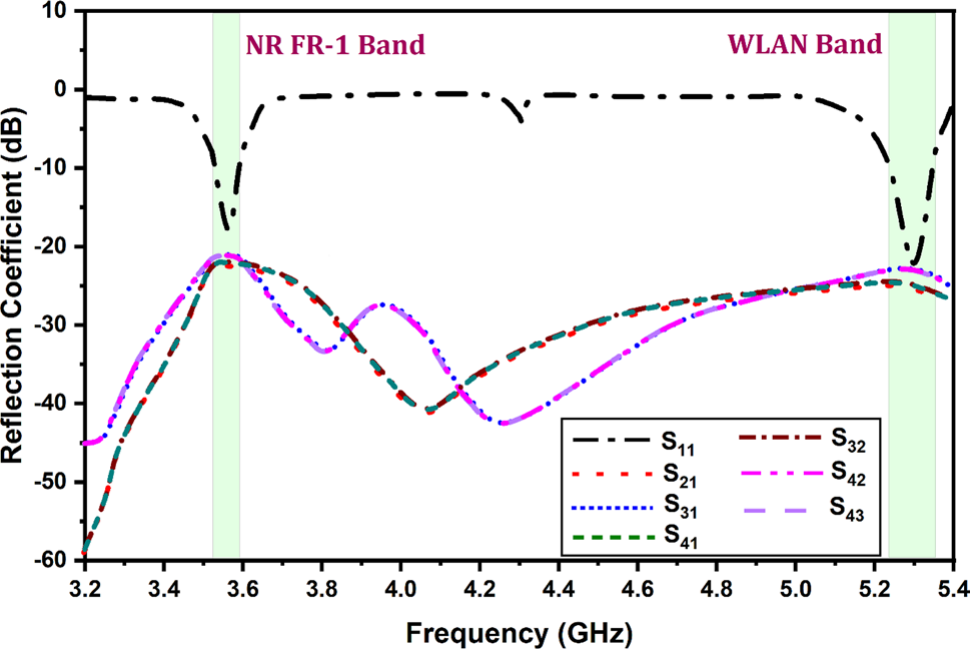
Variation in the reflection coefficient for the simulated antenna structure.

**Figure 8:**
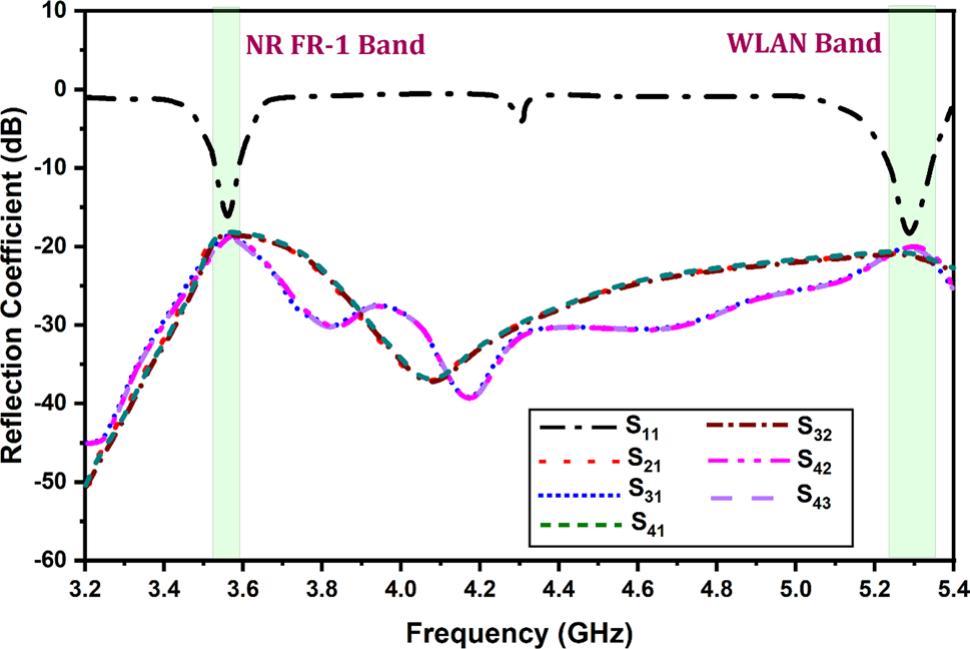
Variation in the reflection coefficient for the measured values of the antenna structure.

Figure 7 shows the variation in the reflectance coefficient for the simulated antenna results for the 3.2–5.4 GHz of the frequency range. It is found that the two operating antenna bands for the NR FR-1 band and WLAN band the minimum return loss values of − 20 dB. It is identified that the other cross-polarization return loss parameters are < − 10 dB for the entire spectrum. Similarly, Fig. 8 shows the variation in the S parameters for the measured results obtained for the proposed MIMO antenna structure.

**Figure 9:**
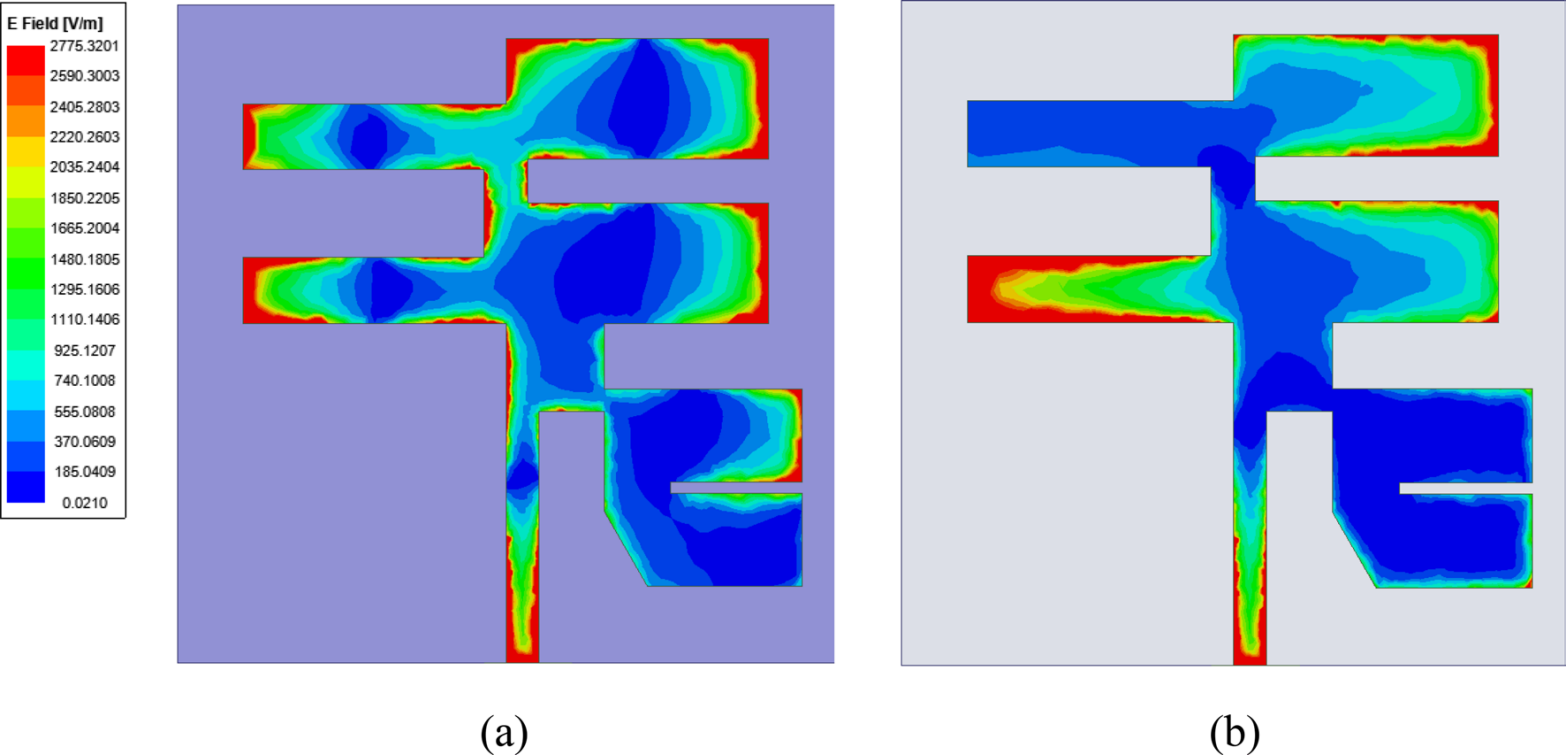
Surface current (**a**) 3.56 GHz and (**b**) 5.28 GHz.

It is found that the measured results are also matched with the simulated structure and show the two similar operating bands of the resonance. There is a minute difference between simulated and measured results. There multiple reasons between simulated and measured values (a) The instrument errors, (b) fabrication tolerances and (c) ideal conditions set by software simulator while numerically computing the parameters. A few of iterations were carried out to confirm the presented results. The surface currents at targeted frequencies are shown in Fig. 9 The relationship between the surface current and the radiation pattern at the operating modes of the rectangle patch. Because of the full reflector at the ground of the antenna, patch radiates more in the exhibited bore sight direction.

**Figure 10:**
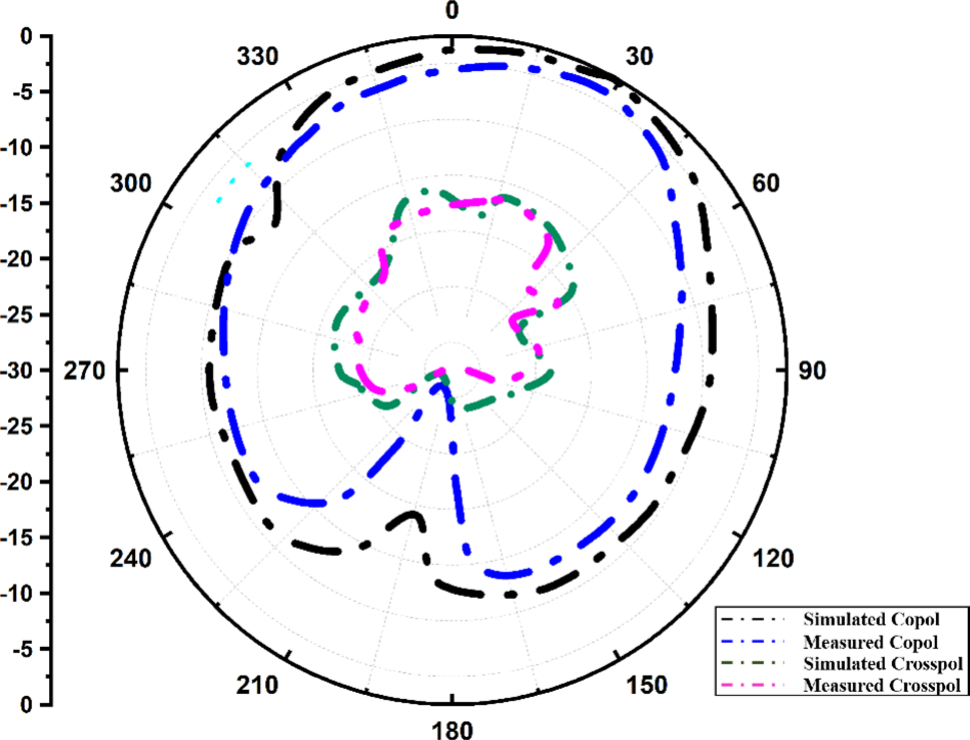
Radiation pattern at 3.56 GHz.

**Figure 11:**
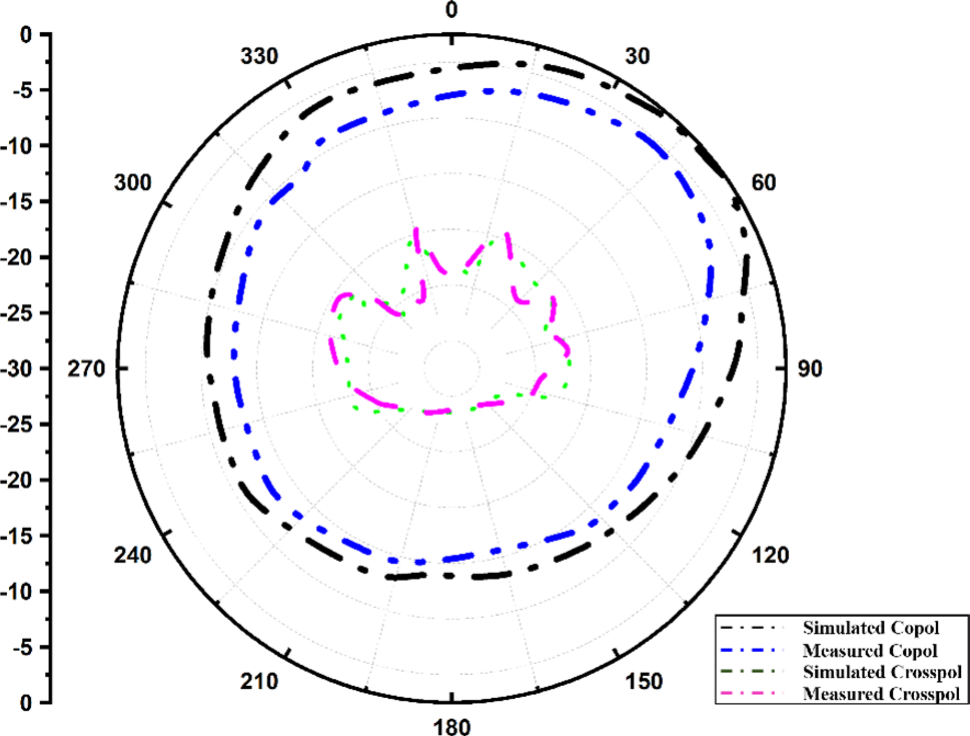
Radiation pattern at 5.28 GHz.

**Figure 12:**
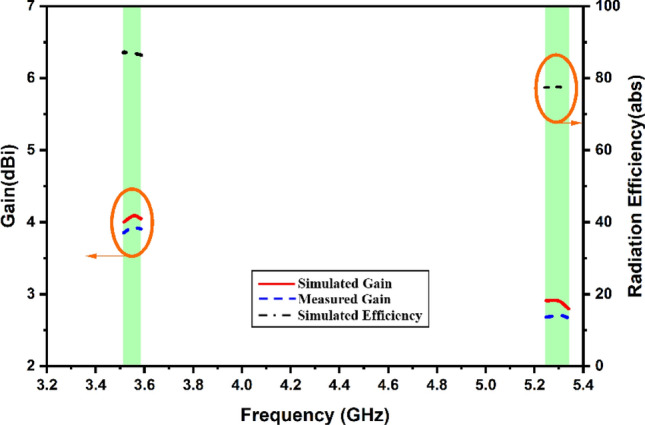
Calculated comparative values of the antenna gain for the specific band of the operation along with antenna efficiency value.

Figures 10 and 11 show the variation in the radiation pattern for the different operating frequencies of 3.56 GHz and 5.28 GHz of the values. Their results are also showing a comparative analysis of the simulated and measured results of the co and cross-polarisation pattern. It is observed that the proposed antenna offers a nearly omni-direction radiation pattern for both resonating frequencies. The measured gain and radiation efficiency of the proposed MIMO antenna are shown in Fig. 12. We have found the ~ 4.2 dBi gain for the first operating band and ~ 2.8 dBi gain for the second operating band with > 80% of the radiation efficiency.

## MIMO antenna parameters

In Eq. (1), ECC is found by first determining the size of the electric field. This is done to calculate ECC. It is common practice to refer to the solid angle components of the ECC using the ith and jth notations. Evaluating ECC requires a large investment of both time and effort since it requires analysing the radiation properties of the far-off area. Equation (3) provides an alternate way of computing ECC^31^. This method makes use of the S-parameters for the calculation of the ECC.

3 $$ {\uprho }_{{{\text{ij}}}} = \left| {\frac{{{\iint }\left( {{\text{E}}_{{{\uptheta }_{{\text{i}}} }} \cdot {\text{E}}_{{{\uptheta }_{{\text{j}}} }}^{*} + {\text{E}}_{{{\varphi }}} :{\text{E}}_{{{{\varphi }}_{{\text{j}}} }}^{*} } \right)^{{{{{\rm d}\Omega }}}} }}{{{\iint }\left( {{\text{E}}_{{{\uptheta }_{{\text{i}}} }} \cdot {\text{E}}_{{{\uptheta }_{{\text{i}}} }}^{*} + {\text{E}}_{{{{\varphi }}_{{\text{i}}} }} \cdot {\text{E}}_{{{{\varphi }}_{{\text{i}}} }}^{*} } \right){{{\rm d}\Omega }}{\iint }\left( {{\text{E}}_{{{\uptheta }_{{\text{j}}} }} \cdot {\text{E}}_{{{\uptheta }_{{\text{j}}} }}^{*} + {\text{E}}_{{{{\varphi }}_{{\text{j}}} }} \cdot {\text{E}}_{{{{\varphi }}_{{\text{i}}} }}^{*} } \right)^{{{{{\rm d}\Omega }}}} }}} \right|^{2} $$

4 $$ {\uprho }_{{{\text{ij}}}} = \frac{{\left| {{\text{S}}_{11}^{*} {\text{S}}_{12} + {\text{S}}_{21}^{*} {\text{S}}_{22} } \right|^{2} }}{{\left( {1 - \left( {\left| {{\text{S}}_{22} } \right|^{2} + \left| {{\text{S}}_{12} } \right|^{2} } \right)} \right)\left( {1 - \left( {\left| {{\text{S}}_{11} } \right|^{2} + \left| {{\text{S}}_{21} } \right|^{2} } \right)} \right)}} $$

**Figure 13:**
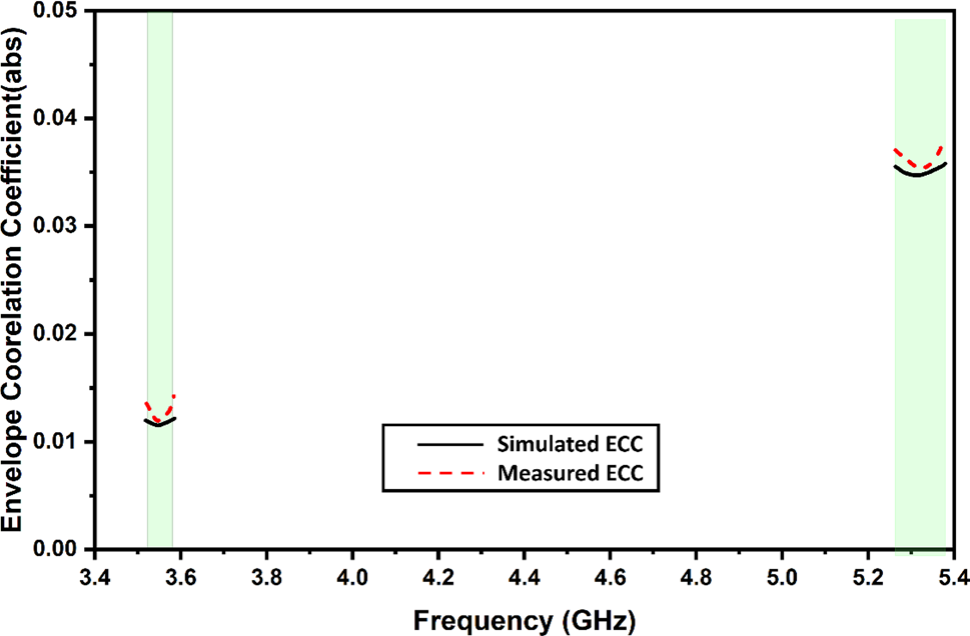
Simulated and measured values of the envelope correlation coefficient.

Calculations for ECC are shown in Fig. 13 for the proposed MIMO antenna structure. Figure 13 illustrates that the ECC of the antenna should be less than 0.05 across the major working band of the antenna. The increased ECC levels are partially responsible for the increased degree of system stability that has been seen. When the ECC value is decreased, the connections between the different antenna components also decrease. Since the numbers are so low, it is presumed that the MIMO performance of the antenna is pretty excellent. Mixing antenna components with unique fading characteristics is one way to reduce the negative impacts of fading.

**Figure 14:**
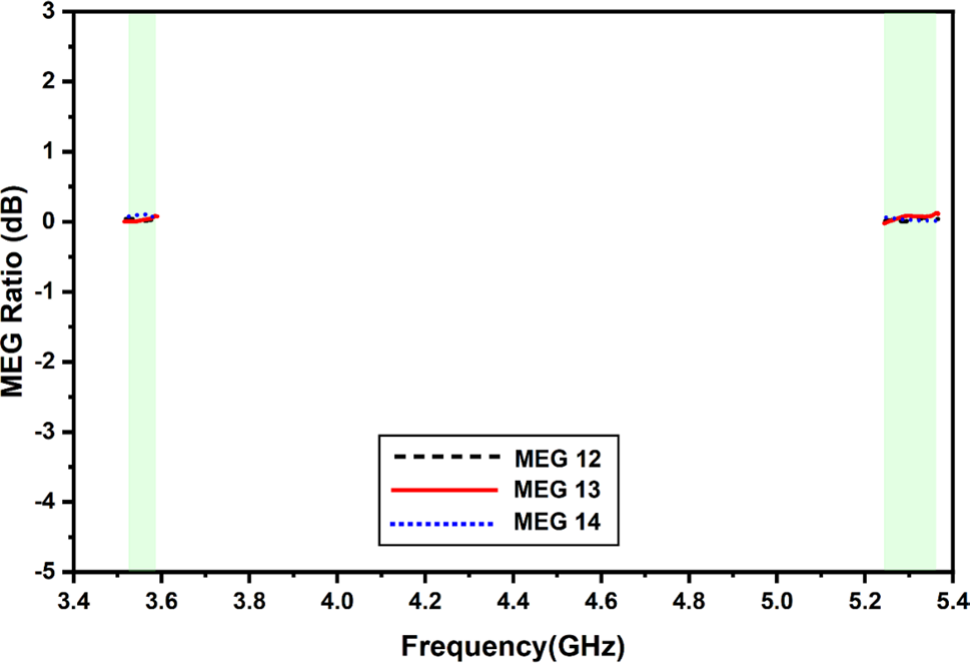
Simulated and measured values of the mean effective gain.

If you put two isotropic antennas at a location where there is no background noise, the total average power that those antennas will receive will either be the same as or less than the average power that a diversity antenna would receive if it is placed in a location where there is background noise (or interference). For instance, the increased performance of a MIMO antenna could be affected by external variables such as the environment in which it is placed. Applying Eq. (5) as recommended in^31^ is feasible to confirm that the MEG is present. As mentioned in^31^, the total number of ports in the current architecture is M. The η denotes the radiation efficiency of the present MIMO structure rad. If you want the optimum performance from the device’s diversity feature across all ports, you must ensure that MEG is set to − 3 dB. In addition to this, the level differences between the two ports must be equal to 0 dB, as shown in Fig. 14.

5 $$ {\text{MEG}}_{{\text{i}}} = 0.5{\upeta }_{{{\text{i}},{\text{ rad}}}} = 0.5\left[ {1 - \mathop \sum \limits_{{{\text{j}} = 1}}^{{\text{M}}} \left| {{\text{S}}_{{{\text{ij}}}} } \right|^{2} } \right] $$

**Figure 15:**
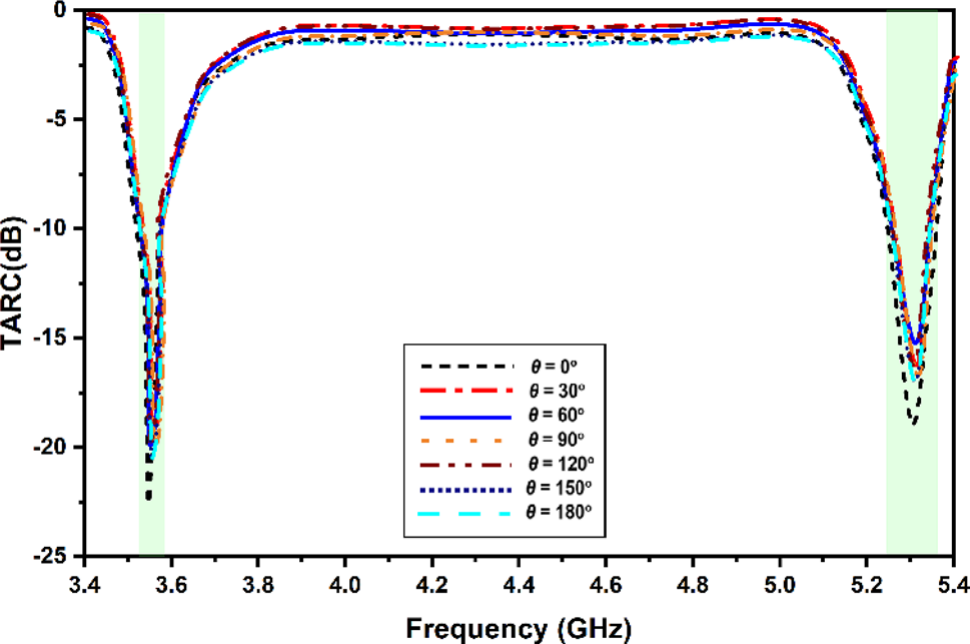
Simulated and measured values of the TARC.

The Total Active Reflection Coefficient, which is more often referred to as simply TARC, is capable of giving an accurate evaluation of radiation performance as well as frequency response when it is applied to a large number of ports. To compute it, take the square root of the total reflected power and divide that amount by the total power that was incident on the item. This will give you the value you're looking for. The solution will be found in the result. The Total Active Reflection Coefficient, sometimes known as TARC, is a statistic that assesses how well a MIMO system bends light. This strategy considers both the random signal pairings that might take place and the mutual coupling that can take place across different networks. It is feasible to analyse the classification of reflected and incident waves using Eq. (6) in the previous sentence. Using Eq. (7) about the S-parameters is the way to retrieve this information, as described in^32,33^. Figure 15 illustrates the variance in TARC values that can be detected between the two distinct configurations of the proposed MIMO antenna. This can be seen by looking at the diagram. It has been found that the performance of data obtained using MIMO in the GHz range is adequate for the purposes for which it is supposed to be used.

6 $${\Gamma }_{{\text{a}}}^{{\text{t}}} = \frac{{\sqrt {\mathop \sum \nolimits_{{\text{j}}}^{{\text{M}}} \left| {{\text{b}}_{{\text{j}}} } \right|^{2} } }}{{\sqrt {\mathop \sum \nolimits_{{\text{j}}}^{{\text{M}}} \left| {{\text{a}}_{{\text{j}}} } \right|^{2} } }} $$

7 $$ {\Gamma }_{{\text{a}}}^{{\text{t}}} = \sqrt {\frac{{\left| {{\text{S}}_{11} + {\text{S}}_{12} {\text{e}}^{{{{{\rm j}\theta }}}} } \right|^{2} + \left| {{\text{S}}_{21} + {\text{S}}_{22} {\text{e}}^{{{{{\rm j}\theta }}}} } \right|^{2} }}{2}} $$

**Figure 16:**
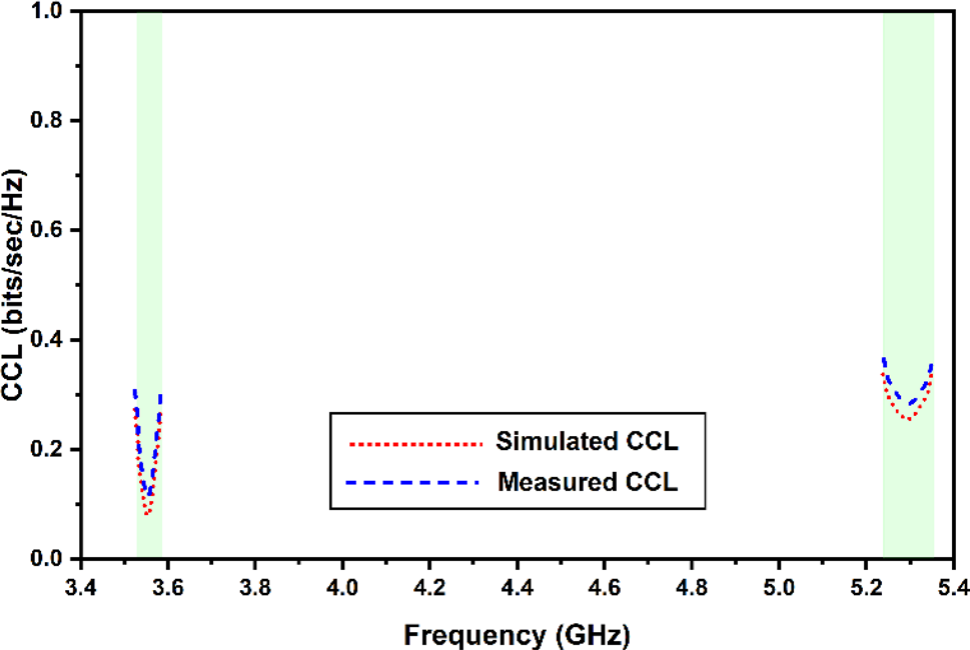
Simulated and measured values of the channel capacity loss.

In assessing the MIMO performance of the chosen THz antenna, the Channel Capacity Loss is an additional significant component that must be considered (CCL). The amount of data that can be sent across a channel at a certain speed without experiencing a substantial loss is the channel’s capacity loss. To successfully signal that information has been sent, the rate must be less than 0.5 bits/second/hertz while using a MIMO system that has been created efficiently. Calculating the CCL parameter may be done using Eqs. (8–10), as stated in^32^. As shown in Fig. 16, this CCL limit has been satisfied for all of the different bands in both setups.

8 $$ {\text{C}}_{{\text{loss }}} = - \log_{2} {\text{det}}\left( {{\text{a}}^{{\text{R}}} } \right) $$

9 $$ {\text{a}}^{{\text{R}}} = \left( {\begin{array}{*{20}l} {{\uprho }_{11} } \hfill & {{\uprho }_{12} } \hfill \\ {{\uprho }_{21} } \hfill & {{\uprho }_{22} } \hfill \\ \end{array} } \right) $$

10 $$ {\uprho }_{{{\text{ii}}}} = 1 - \left( {\left| {{\text{S}}_{{{\text{ii}}}} } \right|^{2} + \left| {{\text{S}}_{{{\text{ij}}}} } \right|^{2} } \right){{, and \rho }}_{{{\text{ij}}}} = - \left( {{\text{s}}_{{{\text{ii}}}}^{*} {\text{S}}_{{{\text{ij}}}} + {\text{s}}_{{{\text{ij}}}}^{*} {\text{S}}_{{{\text{ij}}}} } \right){\text{, where i}},{\text{j}} = 1{\text{ or }}2 $$

**Figure 17:**
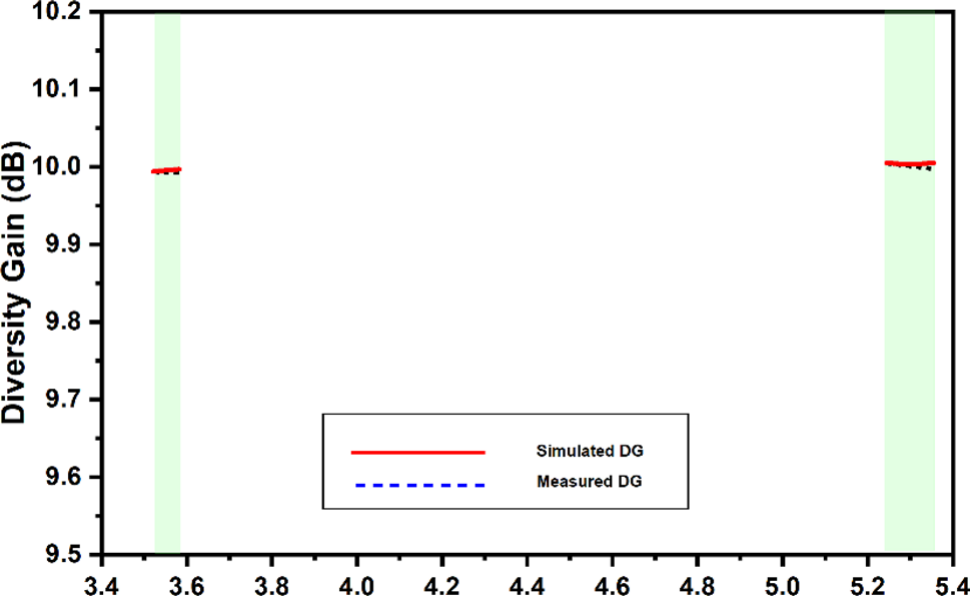
Simulated and measured values of the directivity gain.

The antenna diversity gain (DG) provides the improvement in signal-to-noise ratio (SNR) when the MIMO antenna diversity scheme is established. In the existence of the MIMO system, the SNR is enhanced and subsequently the signal reception. This improves the reliability of the communication system. The DG can be computed from calculated values of ECC as shown in Eq. (5) the values are depicted in Fig. 17.

11 $$ DG = 10\sqrt {1 - \left| {0.99 ECC} \right|^{2} } $$

The antenna is compared with other antennas in the literature to show its utilization in targeted frequencies as exhibited in Table 2.

**Table 2:** Comparison of the proposed antenna with literature.

References	Operating frequencies (GHz)	Antenna size (λ)	Gain (dBi)	Bandwidth (%)	Isolation	ECC	Methodology used
^34^	2.4	1.5 × 1.57	7.95	7.083	> 30 db	0.02	Single pentagonal patch antenna with probe-feed
^35^	3.5, 6, 10	0.45 × 0.45	4.7	117.3	> 15 dB	0.03	Orthogonal circular slots
^36^	3.6	0.75 × 0.75	4.5	0.4	> 19	< 0.12	Hexagonal wide slot ground plane
^37^	4.81	1.1 × 0.45	1.65	6.65	> 15	0.02	Transparent Plexiglas substrate
^38^	29	1 × 0.5	6	3.7	36	0.01	Slits in the radiating patch
^39^	1.9, 5.9	0.114 × 0.114	4.002	1.27	> 19	0.06	Ring and Loop slots in patch
^40^	3.5, 5.5	0.98 × 0.98	5.25	0.12	> 18–20	0.03	Ring DRA
Proposed antenna	3.56, 5.28	1.06 × 1.06	4.2, 2.8	1.82, 2.8	> 22 dB	0.04	Stub and Slots in patch

## Conclusion

We show a four-port MIMO antenna that has a high level of isolation. The antenna's primary purpose is to cover the n48 band of Frequency Range-1 (FR-1), which operates in TDD duplex mode. After achieving the size reduction of a single antenna unit by optimizing the placement of slots and extended arms, the quad-antennas are arranged orthogonally to create antenna diversity. The antenna has resonant frequencies of 3.56 GHz and 5.28 GHz, with a 2:1 VSWR fractional bandwidth of 1.82% and 2.12%, respectively. The effectiveness of the suggested resonator is 88.34% for lower bands and 79.28% for higher bands, accordingly. Due to the excellent inter-element isolation, the antenna performs very well as a radiator regarding MIMO diversity performance. The figures for the envelope correlation coefficient are − 0.005, and the channel capacity loss is 0.1 bits/sec/Hz. The overall active reflection coefficient is − 24.26. High directivity and cross-pol isolation are both helped by the ground plane's complete profile. The antenna has a gain of 4.2 dBi and, accordingly, 2.8 dBi, which is sufficient to meet the requirements of the intended applications. Reported results in the proposed antenna strongly apply to the device used in 5G and WLAN Communication.

## Data Availability

All data generated or analysed during this study are included in this published article.
